# DDT, global strategies, and a malaria control crisis in South America.

**DOI:** 10.3201/eid0303.970305

**Published:** 1997

**Authors:** D R Roberts, L L Laughlin, P Hsheih, L J Legters

**Affiliations:** Uniformed Services University of the Health Sciences, Bethesda, Maryland 20814, USA. droberts@usuhsb.usuhs.mil

## Abstract

Malaria is reemerging in endemic-disease countries of South America. We examined the rate of real growth in annual parasite indexes (API) by adjusting APIs for all years to the annual blood examination rate of 1965 for each country. The standardized APIs calculated for Brazil, Peru, Guyana, and for 18 other malaria-endemic countries of the Americas presented a consistent pattern of low rates up through the late 1970s, followed by geometric growth in malaria incidence in subsequent years. True growth in malaria incidence corresponds temporally with changes in global strategies for malaria control. Underlying the concordance of these events is a causal link between decreased spraying of homes with DDT and increased malaria; two regression models defining this link showed statistically significant negative relationships between APIs and house-spray rates. Separate analyses of data from 1993 to 1995 showed that countries that have recently discontinued their spray programs are reporting large increases in malaria incidence. Ecuador, which has increased use of DDT since 1993, is the only country reporting a large reduction (61%) in malaria rates since 1993. DDT use for malaria control and application of the Global Malaria Control Strategy to the Americas should be subjects of urgent national and international debate. We discuss the recent actions to ban DDT, the health costs of such a ban, perspectives on DDT use in agriculture versus malaria control, and costs versus benefits of DDT and alternative insecticides.


### DDT, Global Strategies, and a Malaria Control Crisis in South America

**Donald R. Roberts, Larry L. Laughlin, Paul Hsheih, and Llewellyn J. Legters**
Uniformed Services University of the Health Sciences, Bethesda, Maryland, USA

Malaria is reemerging in endemic-disease countries of South America. We examined the rate of real growth in annual parasite indexes (API) by adjusting APIs for all years to the annual blood examination rate of 1965 for each country. The standardized APIs calculated for Brazil, Peru, Guyana, and for 18 other malaria-endemic countries of the Americas presented a consistent pattern of low rates up through the late 1970s, followed by geometric growth in malaria incidence in subsequent years. True growth in malaria incidence corresponds temporally with changes in global strategies for malaria control. Underlying the concordance of these events is a causal link between decreased spraying of homes with DDT and increased malaria; two regression models defining this link showed statistically significant negative relationships between APIs and house-spray rates. Separate analyses of data from 1993 to 1995 showed that countries that have recently discontinued their spray programs are reporting large increases in malaria incidence. Ecuador, which has increased use of DDT since 1993, is the only country reporting a large reduction (61%) in malaria rates since 1993. DDT use for malaria control and application of the Global Malaria Control Strategy to the Americas should be subjects of urgent national and international debate. We discuss the recent actions to ban DDT, the health costs of such a ban, perspectives on DDT use in agriculture versus malaria control, and costs versus benefits of DDT and alternative insecticides.

Malaria is reemerging in most disease-endemic countries of South America (Figure 1). Even though disease incidence is increasing, the level of increase is undefined. More importantly, the reasons for the increase in malaria rates after decades of successful disease control have not been assessed. Herein we will show how rapidly malaria is increasing, examine the patterns of resurgent malaria in relationship to the Global Malaria Control Strategy (1), and test the hypothesis that increased malaria is due to decreased spraying of homes with DDT. Also, we will discuss recent actions to ban DDT, the health costs of such a ban, perspectives on DDT use in agriculture versus malaria control, and costs versus benefits of DDT and alternative insecticides.

Address for correspondence: Donald R. Roberts, DPMB, USUHS, 4301 Jones Bridge Rd., Bethesda, MD 20814; fax: 301-295-3860; e-mail: droberts@usuhsb.usuhs.mil.

### Revised Estimates of Malaria Rates in South America

The Pan American Health Organization has been compiling and reporting malaria data since 1959. These data are used to compute the annual parasite index (API) and the annual blood examination rate (ABER). API is the number of positive slides per 1,000 population. For each country, API can be viewed as a measure of numbers of cases detected and numbers of cases treated. API is based on composite data derived from both active and passive case detection; e.g., 63% of all blood slides taken in the Americas during 1994[1] were from passive case detection (2).

API is commonly used to compare amounts of malaria within geographically or temporally distinct human populations. The formula for

[1] This percentage was obtained by adding the number of slides from passive case detection reports and from voluntary collaborators and then dividing the sum by the number of slides acquired through active case detection.

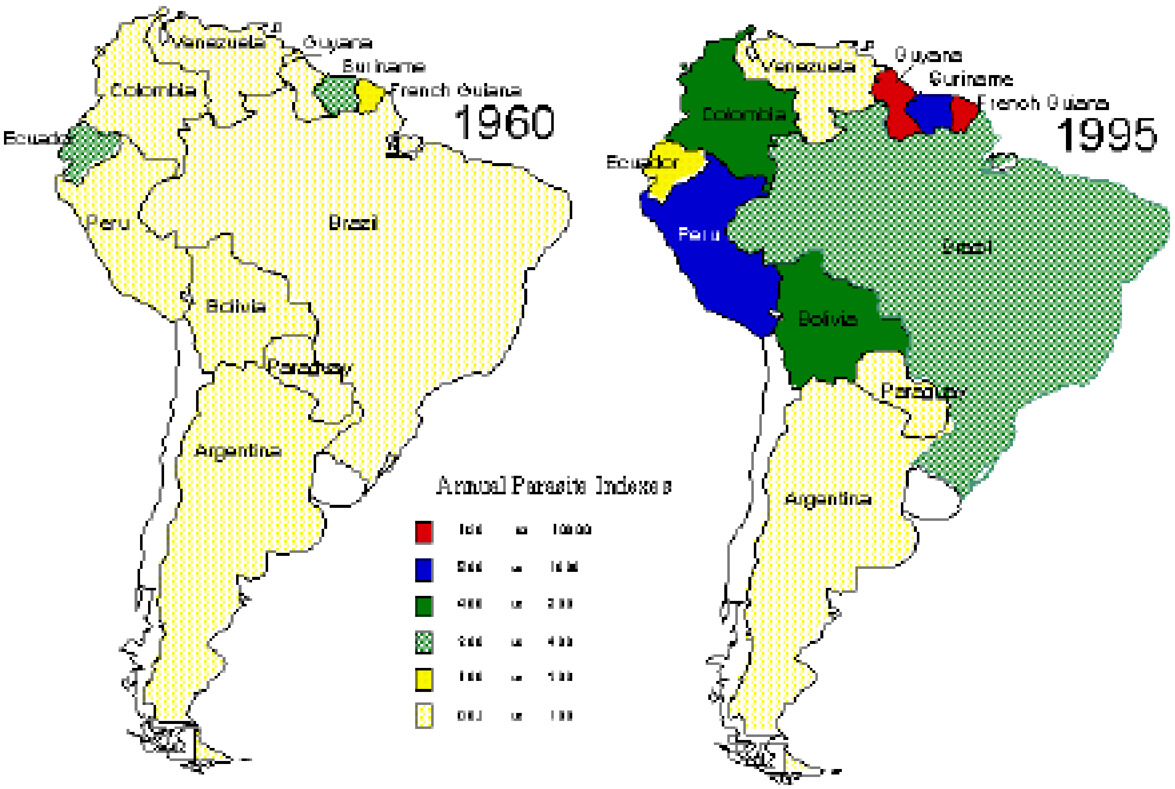

Figure 1. Distribution of malaria in South America (2-5). Color codes correspond to annual parasite indexes as reported by the Pan American Health Organization.

calculating API is

API for year x = 1,000 (number of positive slides/total population)

The number of positive slides (numerator of the API formula) for a given year is a function of the slide positivity rate (which indicates intensity of malaria within the environment) and the total number of slides examined. The total number of slides examined is used to calculate ABER, which indicates case detection effort. The formula for calculating ABER is

ABER for year x = 100 (number of slides examined/total population)

ABER represents the number of slides examined per 100 population.

The general pattern presented by conventional APIs for Brazil (2-4) is presented in Figure 2. Our analysis shows that population growth, combined with decreased numbers of slides examined, underestimates upward trends in malaria cases.

The population of Brazil has grown continuously (Figure 2). The number of blood slides examined each year increased from 1965 to

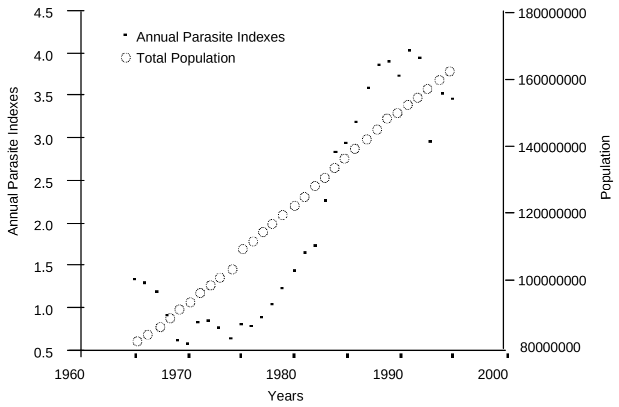

Figure 2. Annual parasite indexes and population growth, Brazil, 1965-1995 (2-5). A graphical representation of data compiled by the Pan American Health Organization.

1985, and then started an erratic decline (Figure 3). Slide positivity rates progressively increased after 1979 (Figure 3). Variable numbers of slides examined, combined with population growth, resulted in ABER values from 2.1 to 2.55 until the mid-1980s; after 1985, ABERs steadily declined (Figure 4).

Given the quantitative components of API, a comparison of indexes is meaningful only if numbers of slides examined relative to the population base are comparable across years. To illustrate, a population of 4,000,000 (with 200,000 slides examined and 60,000 malaria-positive slides) equates to a 30% slide-positivity rate and an API of 15. However, if 400,000 slides are examined, the API will be 30. This example shows how sensitive API is to the number of slides examined.

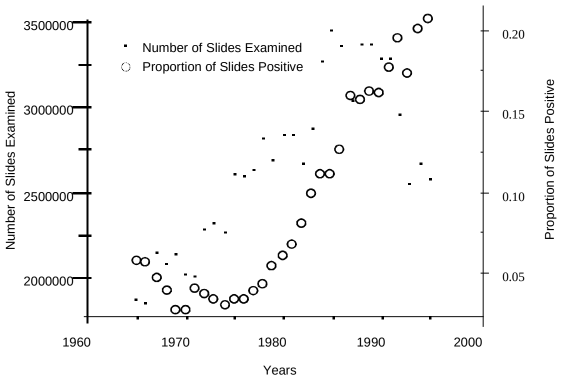

Figure 3. Slides examined and proportions positive for malaria, Brazil, 1965–1995 (2-5). Points correspond to data compiled by the Pan American Health Organization.

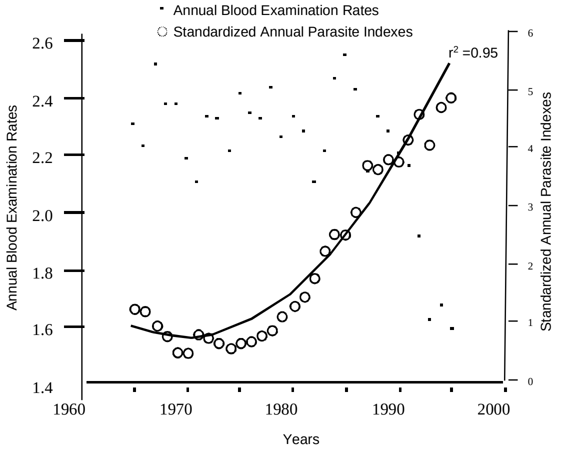

Figure 4. Annual blood examination rates and standardized annual parasite indexes, Brazil, 1965–1995 (2-5). The standardized APIs were adjusted to a common sample size across years (the annual blood examination rate of 1965). Original data for calculating the standardized APIs were obtained from Pan American Health Organization reports (2-5).

We recalculated APIs for Brazil by using the slide-positivity rate for each year and a standardized ABER of 2.31 (ABER for Brazil in 1965). The number of slides examined for each year was recalculated as follows:

Number of slides examined/year = (2.31/100)(total population)

Using this formula for each year, we multiplied the original proportion of positive slides for each year by the revised estimate of total number of slides examined. This calculation provided a uniform estimate of malaria-positive slides. We then divided the estimate of malaria-positive slides by the population of Brazil for each year in the series. These quotients, multiplied by 1,000, produced APIs that were standardized for sampling effort (ABER).

When the derived or standardized API is plotted (Figure 4), the pattern of increasing quantities of malaria is very different from Figure 2. The pattern in Figure 4 shows stable or small yearly increases in malaria rates from 1965 to the late 1970s. After 1978, APIs increased fivefold through 1995. Similar relationships were found with years versus standardized indexes for falciparum and vivax malarias (not shown). Standardized APIs were also developed for Peru and Guyana (2-4). Like Brazil, Peru and Guyana show geometric growth in numbers of malaria cases (Figure 5)(also for falciparum and vivax malarias [not shown]).

It might be argued that the appearance of increasing malaria is an artifact of increasing reliance on passive case detection. Such an argument is equally valid against conventional APIs since the sources of blood smears are the same for both the conventional and standardized indexes. We concede that increased use of passive case detection contributes to increasing slide-positivity rates; however, passive case detection is not sufficient to account for the magnitude or consistency of increases described in Figures 4

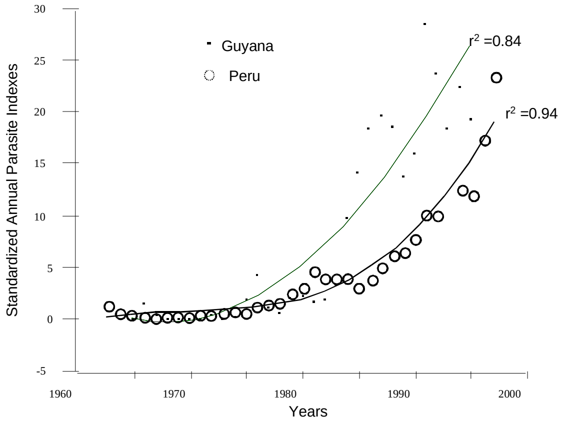

Figure 5. Standardized annual parasite indexes, Peru (1959–1995) and Guyana (1960–1995). The original data were derived from Pan American Health Organization reports (2-5). The APIs were adjusted to a common sample size across years (the annual blood examination rate of 1965).

and 5. Using Brazil as an example, more than 35% of slides were taken through passive case detection in 1972.[2] In 1991, 32.8% of all slides in Brazil were obtained through passive case detection (5). Clearly, the 17-year pattern of increasing malaria is not due to increased reliance on passive case detection.

### The Global Malaria Control Strategy

The policies and strategies of the World Health Organization, the Pan American Health Organization, and national donor agencies contributed to the successful control of malaria from the late 1940s to the late 1970s (6-8). However, the policies and strategies of these organizations have changed (8). In 1979, the World Health Organization Expert Committee on Malaria (9) developed a new malaria control strategy with four tactical variants. Variants 1 and 2 included no organized vector control measures. Variant 3 included limited vector control, and only variant 4 included, for highly qualified countries, countrywide vector control. The goal for variant 4 was specified as eradication, not sustained malaria control. The new strategy was adopted by the 31st World Health Assembly as resolution WHA 31.45 (9). During this assembly, the Director-General of World Health Organization stressed the importance of including curative and preventive services, including control of infectious diseases (malaria control), in the framework of primary health care. In 1985, the 38th World Health Assembly adopted resolution WHA 38.24, which recommended that malaria control be developed as an integral part of the national primary health care system (8). In October 1992, the Ministerial Conference adopted the Global Malaria Control Strategy that had been developed at World Health Organization interregional meetings in 1991 and 1992 (1). The Global Malaria Control Strategy calls for deemphasis of vector control and emphasizes case detection and treatment. The first conclusion and recommendation of the report is that malaria control should be fully integrated into general health services and should reflect socioeconomic development objectives. The World Health Assembly resolutions and committee reports document, from 1979 to the present, general, and sometimes specific, policy decisions that promote case detection and treatment and deemphasize residual spraying for national malaria control programs.

Failure to maintain control over malaria most likely results from failures in the functions of interventions or from failures to make proper application of interventions. Although DDT resistance is often posed as a reason for malaria control failure, resistance of vector populations to DDT is not widespread in South America (10).

There are two common interventions for reducing malaria transmission within human populations. One potential intervention is case detection and treatment. The second is spraying insecticide on house walls to prevent malaria transmission inside the houses.

### DDT and the Reemergence of Malaria

Field studies of DDT action against malaria vectors provide dramatic evidence of reduced human-vector contact (11-13). However, the effect of DDT against human malaria cannot be definitively answered by vector studies alone. Consequently, we studied the effect of DDT on malaria rates with regression models to look at the interactive effects of home-spray and malaria rates across years of malaria control activities.

[2] Statistics drawn from a U.S. Agency for International Development review in 1973-1974 of the malaria eradication program in Brazil and based on regions that were in the attack phase. There was greater reliance on active and epidemiologic surveys in such areas than in areas of consolidation or maintenance. Therefore, the overall percentage of slides derived from passive case detection was undoubtedly higher than 35%.

Once missing data were factored into the analyses, 28 and 32 years of malaria control data were used in separate tests for Brazil and Ecuador, respectively. Brazil was selected because it has sustained a robust malaria control program with progressive decreases in numbers of houses sprayed with DDT (Figure 6). Ecuador also reported progressive decreases in numbers of houses sprayed with DDT. However, Ecuador also reported great vacillations in spray rates (Figure 6), which were accompanied by variations in malaria rates. Data presented in Pan American Health Organization reports (2-5) were used in these analyses; i.e., malaria rates were not standardized by a fixed ABER.

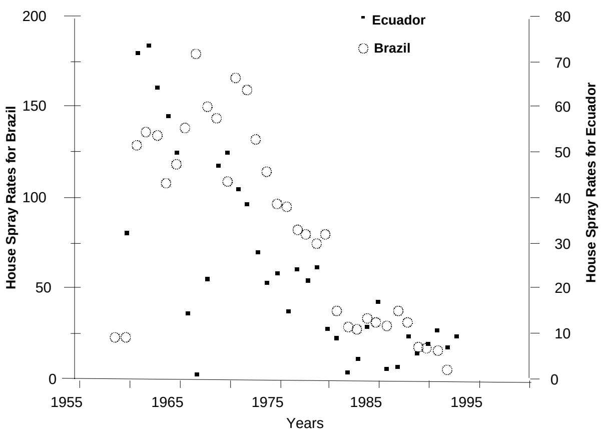

Figure 6. House-spray rates (HSRs) with DDT, Brazil and Ecuador, 1959–1993. Points correspond to data compiled by the Pan American Health Organization (2-5).

Preliminary tests with a normalized API variable as the dependent variable and house-spray rates (HSRs), time, and ABER as independent variables showed statistically significant negative relationships between HSR and API values. However, the normalized response variables did not fulfill the requirement of constant variance, and the model exhibited problems of autocorrelation.

The problems with data variance and autocorrelation were solved for Brazil data by performing analyses with the following regression model:

$$\gamma_t = \alpha + \beta_1 \gamma_{t-1} + \beta_2 X_{t-1} + \varepsilon$$

The $\gamma$ represents an API value and t is time (year). Given that $\gamma_t$ represents API for year t, then $\gamma_{t-1}$ represents API for the preceding year. This component of the formula represents a lag year API value. The $\alpha$ is intercept, and ß is a parameter (slope) of the $\gamma$ regression line. The HSR is from the year preceding $\gamma_t$, represented by $X_{t-1}$. Last, $\beta_2$ is slope of the HSR regression line, and $\varepsilon$ is the residual effect not accounted for by the API and HSR variables. This formula captures the idea that API plus HSR of one year can be used to accurately predict the API of the following year.

The analysis of variance of 28 years of data from Brazil produced an excellent fit of the regression model ($F = 354$; df=(2,26); $p < 0.0001$). The adjusted $r^2$ for the regression analysis was 0.96. Parameter estimate for $\gamma_{t-1}$ was 0.74, and this relationship was highly significant ($p < 0.0001$). The parameter estimate for $\mathbf{X}_{t-1}$ was -0.0174; it was also highly significant ($p < 0.0004$). A test for autocorrelation was performed by correlation analysis of residuals versus lag time residuals. The Pearson Correlation Coefficient test sta-tistic was used to test the null hypothesis that $\rho$ =O. The p value was 0.87 (not statistically significant), so we accepted the null hypothesis of no autocorrelation.

The problems of variance and autocorrelation were solved for data from Ecuador by performing regression analyses with the following model:

$$\gamma_t = \alpha + \beta_1 \gamma_{t-1} + \beta_2(\log(X_{t-1})) + \varepsilon$$

The only difference between the Brazil and Ecuador models was a log transformation of the lag HSR values. A log transformation of lag HSR values reduced variation in this variable and produced a better fit of the whole model.

The regression analysis of 32 years of data from Ecuador produced an excellent fit of the regression model ($F = 45.6$; df=(2,30); $p < 0.0001$). The adjusted $r^2$ for the regression analysis was 0.73. Parameter estimate for $\gamma_{t-1}$ was 0.58, and this relationship was highly significant ($p < 0.0001$). The parameter estimate for $\log(X_{t-1})$ was -1.197; it was also highly significant ($p < 0.0001$). The test for autocorrelation produced a p value of 0.51 (not statistically significant), and again we accepted the null hypothesis of no autocorrelation.

These highly predictive models showed the powerful relationship between DDT-sprayed houses and malaria rates. We documented that a high API in combination with a high HSR is predictive of a lower API the following year. Alternatively, when low APIs are combined with low HSRs, malaria rates are higher the following year.

Therefore, when large numbers of houses are sprayed with DDT, malaria rates decline and when fewer houses are sprayed, malaria rates increase.

### Eliminating DDT for Malaria Control

Countries are banning or reducing the use of DDT because of continuous international and national pressures against DDT (e.g., the International Pesticide Action Network is "...working to stop the production, sale, and use..." of DDT [14]) and aggressive marketing tactics of producers of more expensive alternative insecticides. It has become easier for political pressures to succeed given the global strategy to deemphasize use of the house-spray approach to malaria control. A recent agreement of the North American Commission on Environmental Cooperation for eliminating the production and use of DDT in Mexico within the next 10 years[3] is the latest development in the campaign to eliminate DDT.

### The Health Costs of Abandoning DDT

There is a cost in abandoning DDT for malaria control. This cost is seen in the results of malaria control programs from 1993 to 1995. We can get a uniform picture of events from 1993 to 1995 by standardizing malaria rates according to size of population at risk for malaria in each country (3,4). Since there were variations in this population variable for the 3 years, we took the population estimates for the midyear interval, 1994, as the basis for adjusting malaria rates for 1993 and 1995 (2). Each country was also characterized according to its reported use of DDT for malaria control in 1993 through 1995 (2-4).

As shown in Figure 7, countries that discontinued their house-spray programs reported large increases in malaria rates. Countries that reported low or reduced HSRs also reported increased malaria. Only Ecuador reported increased use of DDT and greatly reduced malaria rates.

### The Use of DDT in Agriculture versus Malaria Control

In 1993, North, Central, and South American countries used 1,172,077 kg of DDT to spray house walls (4). While this may seem to be a large

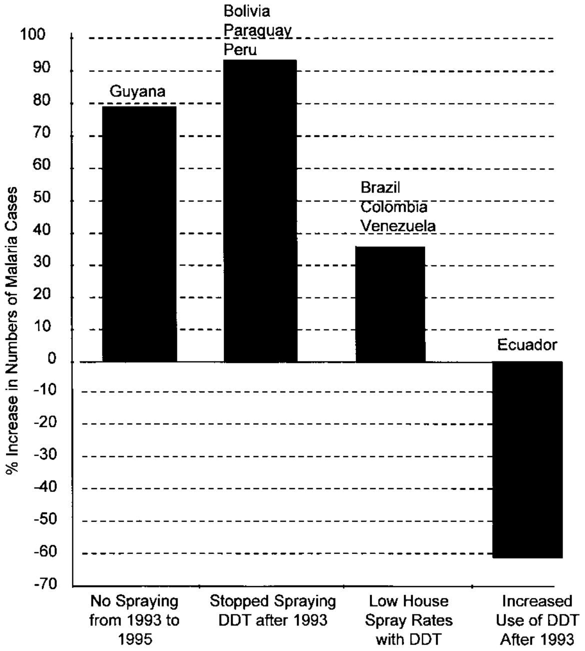

Figure 7. Increases in annual parasite indexes for four categories of countries, South America, 1993–1995. For each country, the populations at moderate to high risk for malaria were adjusted to midyear (1994) values. Data were derived from reports of the Pan American Health Organization (2-5).

amount of insecticide, it actually represents less than 6% of the DDT used in the United States alone in 1968 (15). More than 795 kg of DDT might be used to treat a mere 0.4 $km^2$ (100 acres) of cotton during a growing season.[4] This amount of DDT would be sufficient to treat more than 1,692 houses. At four to five persons per house, spraying 1,692 houses translates into protection for as many as 8,460 persons. Since rural households are the primary candidates for house spraying, the 1,692 houses would be spread over a very large area. If a household of five persons is used, for example, significant levels of malaria control could be obtained for all populations at moderate to high risk for malaria transmission in Guyana by spraying only 17,000 houses during a single spray cycle. This level of treatment for the whole country of Guyana, covering an area of 215,000 $km^2$, is roughly equal to the amount of

[3]The Commission for Environmental Cooperation (CEC) is a North American environment commission established by a North American Free Trade Agreement side agreement. The CEC draft agreement entitled "North American Regional Action Plan on DDT, Task Force on DDT and Chlordane," dated October 10, 1996, calls for the elimination, distribution, and use of DDT for malaria control in Mexico in 10 years.

[4]Recommended weekly treatments of 0.9-1.36 kg (2-3 pounds) of DDT per 0.004 $km^2$ (1 acre) of cotton. Using a 7-week period and a treatment of 1.13 kg (2.5 pounds) per 0.004 $km^2$, 340 kg (1.750 pounds) of DDT is required for 0.4 $km^2$ (100 acres) of cotton.

DDT that might be used to spray only 4 $km^2$ (1,000 acres) of cotton during a single growing season. These statistics demonstrate the differences between DDT for agriculture and DDT for malaria control. On a landscape scale, a sprayed house represents an infinitesimally small spot treatment of a closed and protected environment (the house). DDT is relatively insoluble in water, so even when a house collapses and decays, DDT will not easily move from the house site.

### Cost versus Benefit

Based on statistics compiled in 1978,[5] costs of chemicals for protecting a person showed malathion to be five times more expensive than DDT. In evaluating the cost of case treatment versus insecticide spraying, it is important to weigh the fact that a treated person will probably return to sleep in the very house where a potentially infectious blood meal was served to malaria vector mosquitoes the night(s) before diagnosis and treatment. Even with treatment and cure, persons can become reinfected by mosquitoes that fed on them before treatment. Indeed, a person can be reinfected and undergo curative treatment repeatedly over a period of a few months. While DDT residues do not provide complete protection from malaria transmission, they do provide variable but significant levels of protection for months after walls are sprayed.

A study of DDT alternatives for malaria control in Ecuador showed that the cost of other insecticides was many times higher than the cost of $1.44 to spray one house per year with DDT (16). The prohibitive cost of DDT alternatives has been a problem in malaria control (16). From 1986 to 1988, Mexico evaluated DDT alternatives in its national malaria program but discontinued their use because of unfavorable responses and high costs (17). High costs and downward trends in foreign aid suggest that many countries cannot afford the switch to DDT alternatives.

In 1994, the U.S. Agency for International Development allocated only $850,000 for malaria control in the Americas, compared with $4.13 million for malaria vaccine research (2). National malaria control budgets in the Americas declined 27% from 1994 to 1995, and loans and grants declined by 29%. With economic downsizing and reduced levels of foreign aid from industrialized countries (3), there is little likelihood that more money will be allocated for more expensive insecticides.

### Discussion and Conclusions

Malaria is reemerging in disease-endemic countries. We have shown the patterns of real growth in malaria rates for Brazil, Peru, and Guyana. Figure 8 shows a similar pattern of growth in malaria rates for 18 other countries of the Americas. Figure 8 also depicts the relationships of increased malaria incidence to changing global strategies for malaria control. There is no inference of causation between changing policies and malaria increases. In fact, the HSRs were declining (as illustrated in Figure 8) even before global strategies were changed. However, it certainly seems that the new strategies are not producing a desirable outcome.

We have used two regression models to show

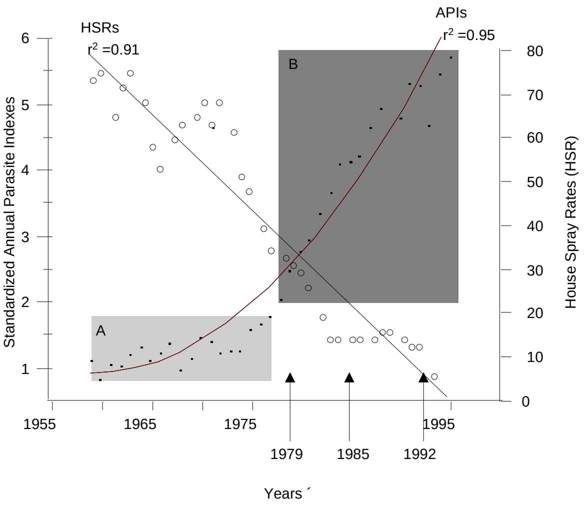

Figure 8. Standardized annual parasite indexes for 21 countries of the Americas, 1959–1995. Major changes in global malaria control strategies are depicted with arrows along the x axis (WHA 31.45 for 1979; WHA 38.24 for 1985; and the Global Malaria Control Strategy for 1992). Statistical data were derived from reports of the Pan American Health Organization (2-4). Block A represents a period of malaria control by spraying adequate numbers of houses with insecticide residues (primarily DDT). Block B represents a period of increasing malaria as the house-spray rates declined below effective levels. Open circles represent house-spray rates and solid squares represent standardized annual parasite indexes.

[5] World Health Assembly document A31/19, 1978.

that as numbers of DDT-sprayed houses declined, malaria incidence increased. The period from 1959 to 1978 can be characterized as a period of insecticide-controlled malaria. The period from 1979 to 1995 can be characterized as a period of decreased use of residual spraying and geometric growth in malaria incidence. Other factors contribute to resurgent malaria, but none would appear to equal the influence of decreases in the house-spray programs.

Public health researchers in the United States helped initiate the use of DDT for malaria control in 1943 (19). Today, DDT is still needed for malaria control. If the pressure to abandon this effective insecticide continues, unchanged or declining health budgets, combined with increasingly expensive insecticides and rising operational costs, will result in millions of additional malaria cases worldwide.

DDT should be produced and distributed for governments to use in malaria control only. Use of this insecticide should not be abandoned unless its known detrimental health effects are greater than the effects of uncontrolled malaria on human health.

The multifaceted issues of DDT use for malaria control (e.g., ecologic damage, human carcinogenicity, and pesticide resistance) and the applicability of the Global Malaria Control Strategy to the Americas should be the subject of intensive national and international debate. We are now facing the unprecedented event of eliminating, without meaningful debate, the most cost-effective chemical we have for the prevention of malaria. The health of hundreds of millions of persons in malaria-endemic countries should be given greater consideration before proceeding further with the present course of action.

Dr. Roberts is professor, Department of Preventive Medicine/Biometrics, Uniformed Services University of the Health Sciences (USUHS), Bethesda, MD. His research focuses on modeling of malaria control methods and on the applications of remote sensing and geographic information systems to malaria control in the Americas.

### References

1. World Health Organization. Implementation of the global malaria control strategy. World Health Organ Tech Rep Ser 1993; no. 839.
2. Pan American Health Organization. Status of malaria programs in the Americas. XLIII Report. Washington (DC): PAHO; 1995.
3. Pan American Health Organization. Status of malaria programs in the Americas. XLIV Report. Washington (DC): PAHO; 1996.
4. Pan American Health Organization. Status of malaria programs in the Americas. XLII Report. Washington (DC): PAHO; 1994.
5. Pan American Health Organization. Status of malaria programs in the Americas. XL Report. Washington (DC): PAHO; 1991.
6. World Health Organization. Sixth Report, Expert Committee on Malaria. World Health Organ Tech Rep Ser 1957; No. 123.
7. Brown AWA, Haworth J, Zahar AR. Malaria eradication and control from a global standpoint. J Med Entomol 1976;13:1.
8. Gilles HM, Warrell DA. Bruce-Chwatt's essential malariology. Boston: Edward Arnold; 1993.
9. World Health Organization. Seventeenth Report, WHO Expert Committee on Malaria. World Health Organ Tech Rep Ser 1979; No. 640.
10. World Health Organization. Resistance of vectors of disease to insecticides. World Health Organ Tech Rep Ser 1980; No. 655.
11. Roberts DR, Andre RG. Insecticide resistance issues in vector-borne disease control. Am J Trop Med Hyg 1994;50:21.
12. Roberts DR, Alecrim WD. Behavioral response of *Anopheles darlingi* to DDT-sprayed house walls in Amazonia. Bull Pan Am Health Organ 1991;25:210-7.
13. Rozendaal JA. Behavioral responses of *Anopheles darlingi* in Suriname to DDT residues on housewalls. J Am Mosq Control Assoc 1989;5:351-8.
14. Pan American Health Organization. PAHO Environmental Series 1993;12:53.
15. Agricultural Stabilization and Conservation Service. Pesticide review. United States Department of Agriculture; 1968.
16. Arellano F. Uso de insecticidas en el programa de control de la malaria del Ecuador. Estrategia aconsejada. In: Comité Rotario Interdistrital Andino de Lucha Contra la Malaria, editors. Nuevas estrategias contra la malaria. Quito, Ecuador: Rotario Interdistrital Andino de Lucha Contra la Malaria; 1990.
17. Bruce-Chwatt LJ. The cost of malaria and of its control in relation to socio-economic realities. In: Study Group on Malaria in the Americas, editors. Washington (DC): Pan American Health Organization, 1977.
18. Pan American Health Organization. Empleo de insecticidas en salud pública Estados Unidos Mexicanos, 1957-1987. In Proceedings of "Taller sobre resistance," PAHO meeting 1988 18-29 Julio, Guatemala.
19. Gahan JB, Travis BV, Morton FA, Lindquist AW. DDT as a residual-type treatment to control *Anopheles quadrimaculatus*, practical tests. J Econ Entomol 1945;38:223-35.
